# Emergency battlefield cricothyrotomy complicated by tube occlusion

**DOI:** 10.4103/0974-2700.44686

**Published:** 2009

**Authors:** Andrew C Gallo, Bruce D Adams

**Affiliations:** 1 Transitional Intern, William Beaumont Army Medical Center, 5005 N. Piedras St El Paso, TX 79920 USA; 2 Department of Clinical Investigations, William Beaumont Army Medical Center, 5005 N. Piedras St El Paso, TX 79920, USA

**Keywords:** Cricothyrotomy, battlefield, airway, occlusion, complications

## Abstract

Emergency cricothyrotomy is a technique used to secure an otherwise compromised or inaccessible airway and has been recommended for use in the battlefield under certain circumstances. This case reports an acute complication of emergency cricothyrotomy. An Iraqi soldier, injured in an improvised explosive device blast received an emergency battlefield cricothyrotomy. At the Combat Support Hospital, the patient became more difficult to ventilate and was taken to the operating room for tracheostomy. The cricothyrotomy tube was found to be occluded with blood.

It is widely accepted that ensuring adequate oxygenation is one of the primary objectives in the management of a trauma patient. Therefore, airway control is the top priority in current algorithms for trauma life support. Although many ways of securing an airway are available and will not be discussed,[1] in certain cases an emergency cricothyrotomy is an important procedure used to secure an otherwise compromised or inaccessible airway. It has been commonly used both in the hospital[2] and by pre-hospital personnel[3,4] with success.

Emergency cricothyrotomy is generally recommended in cases where providers are unable to intubate the trachea with an endotracheal or nasotracheal tube[5] such as severe oropharyngeal hemorrhage, edema of the glottis, fracture of the larynx,[1] and in some patients with C-spine trauma.[6] In a battlefield environment, it has been recommended that emergency cricothrotomy be considered early because battlefield medics may not be able to intubate trauma patients successfully.[7]

Reported short-term complications of emergency cricothyrotomy include misplacement of the airway, subsequent need for chest tube, bleeding,[2] laryngeal fracture,[8] incision error,[9] and thyroid cartilage injury.[10] We encountered a case which illustrates an additional complication that has not been reported in the literature to our knowledge. A young, male Iraqi soldier was injured in an improvised explosive device blast, sustaining multiple blast and shrapnel wounds to his right leg, torso, and face. He lost consciousness and was initially cared for by a US Forward Surgical Team (FST) consisting of a general surgeon, a combat medic, and a nurse anesthetist (CRNA). As part of the primary survey, it was determined that the patient needed an urgent, definitive airway, and a CRNA attempted to intubate the patient with an endotracheal tube by direct laryngoscopy. The endotracheal tube was passed and the team attempted to ventilate the patient's lungs. However, an endotracheal position could not be confirmed by physical examination or color change on the end-tidal CO_2_ indicator. A general surgeon performed an emergency cricothyrotomy and a 6-0 standard ETT was placed and secured with the balloon inflated. The FST further stabilized the patient and transported him to a nearby Combat Support Hospital for more definitive care. Upon arrival to the Combat Support Hospital, the cricothyrotomy tube appeared to be providing adequate ventilation as indicated by auscultation of bilateral breath sounds and the presence of end-tidal CO_2_ by color change. However, during the continued management of this patient's injuries he became progressively difficult to ventilate. His oxygen saturation dropped from 96% to less than 90%, and subsequently the patient went to the operating room for a tracheostomy and a right below knee amputation. When the 6-0 ETT was removed, it was found to be occluded with blood [Figure 1, Figure 2]. A formal tracheostomy was performed and provided adequate ventilation for the remainder of the patient's course. The patient survived his injuries and left the hospital nine days later in satisfactory condition.

**Figure 1:**
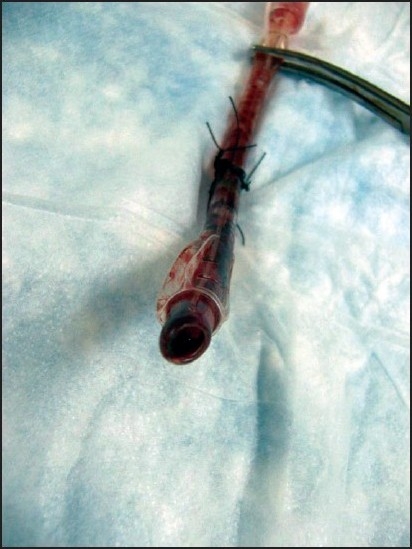
6-0 endotracheal tube used in emergency cricothyrotomy occluded with blood

**Figure 2:**
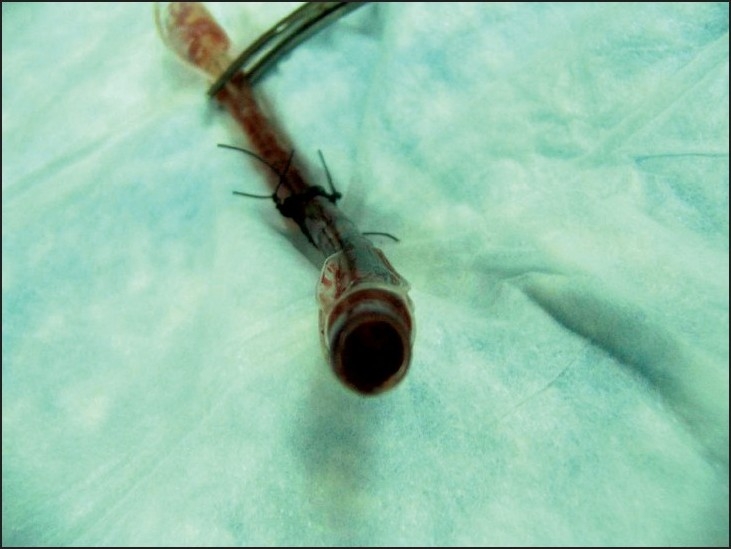
6-0 endotracheal tube occluded with blood

Emergency battlefield cricothyrotomies are an important procedure in the care of a trauma patient. Although acute complications are possible, to our knowledge acute tube occlusion has not been reported in the literature. Clinicians should be aware of this possible complication and should be ready for emergent replacement over a Bougie or a formal tracheostomy. It is important to note that a 6-0 ETT was used in the cricothyrotomy and may have been too small for the patient.[6] Further study to determine the rate of occlusion between comparably sized standard ETT and Shiley tubes in bloody field would be worthwhile.
